# High-Resolution Melting (HRM) of the Cytochrome B Gene: A Powerful Approach to Identify Blood-Meal Sources in Chagas Disease Vectors

**DOI:** 10.1371/journal.pntd.0001530

**Published:** 2012-02-28

**Authors:** Victor H. Peña, Geysson J. Fernández, Andrés M. Gómez-Palacio, Ana M. Mejía-Jaramillo, Omar Cantillo, Omar Triana-Chávez

**Affiliations:** Grupo Biología y Control de Enfermedades Infecciosas (BCEI), Sede de Investigación Universitaria, Universidad de Antioquia, Medellín, Colombia; International Centre of Insect Physiology and Ecology, Kenya

## Abstract

Methods to determine blood-meal sources of hematophagous Triatominae bugs (Chagas disease vectors) are serological or based on PCR employing species-specific primers or heteroduplex analysis, but these are expensive, inaccurate, or problematic when the insect has fed on more than one species. To solve those problems, we developed a technique based on HRM analysis of the mitochondrial gene cytochrome B (Cyt b). This technique recognized 14 species involved in several ecoepidemiological cycles of the transmission of *Trypanosoma cruzi* and it was suitable with DNA extracted from intestinal content and feces 30 days after feeding, revealing a resolution power that can display mixed feedings. Field samples were analyzed showing blood meal sources corresponding to domestic, peridomiciliary and sylvatic cycles. The technique only requires a single pair of primers that amplify the Cyt b gene in vertebrates and no other standardization, making it quick, easy, relatively inexpensive, and highly accurate.

## Author Summary

Chagas disease is one of the most important tropical diseases in America. This disease is caused by the parasite *Trypanosoma cruzi* and transmitted through the feces of blood-sucking insects known as triatomines. Different species of insects have different habits and food sources that confer variable degrees of epidemiological importance. In this paper, we propose the use of High Resolution Melting (HRM) analysis of cytochrome b (cyt b) gene PCR products to identify blood-food sources in triatomines. This tool can effectively differentiate blood-meal sources of insects collected from the field. Such data allows for targeted investigations of insect species that are likely to be involved in the transmission of the parasite to humans in different regions. This knowledge is very important because it allows establishing and designing vector control and surveillance strategies according to each geographical area and to stop the transmission of the parasite to human populations by insects.

## Introduction

*Trypanosoma cruzi*, the etiological agent of Chagas disease, is transmitted by hematophagous Reduviidae insects belonging to Triatominae subfamily, which are mostly distributed on the American continent and comprises 141 species in 15 genera [1], [2]. *T. cruzi* can infect more than 180 species of terrestrial and arboreal mammals belonging to nine orders and 25 subfamilies, which are natural blood-meal sources of triatomine bugs [3].

Depending on the habitats of triatomines, at least two transmission cycles of *T. cruzi* have been identified: domestic and sylvatic. The first one includes humans, domestic animals, and domiciliated vectors. The second one involves sylvatic insects and wild animals [4]. A third cycle associated with anthropic environments can occur in several places where domestic animals are kept in sheds near dwellings, called by some authors a peridomestic cycle [5]. However, the epidemiological scenario of Chagas disease is more complex because it can include overlapping cycles [6]–[10], intrusions of wild vectors into human dwellings [11], [12], colonization and re-infestation of new sylvatic vector species after spraying houses [4], [13]–[15], the regular intrusion of wild mammals inside houses [16], house infestation by two vector species [17], etc.

The identification of *T. cruzi*'s transmission cycle has been considered an essential issue to establish and design vector control and surveillance strategies [18]. In this sense, the determination of blood-meal sources in triatomine insects is essential to identify infection foci, risk factors, and new vector and mammal species involved in the transmission cycles [19]. In triatomines, so far, the methods used for this purpose include serologic and PCR-based molecular techniques. ELISA-based serological techniques are the most frequently used in blood-meal determination of Chagas disease vectors [20]–[24]. However, this method lacks specificity because it cross-reacts with species within the same family and is time-consuming and very expensive because it requires the preparation of specific antisera for each vertebrate host involved [25].

Unlike serological tools, PCR-based molecular methods are more specific and usually easier to perform, although the design of specific primers for each species involved in transmission scenarios makes it difficult to identify the vector and mammals from the wild cycle where a wide variety of species exist. Additionally, this technique is wasteful because each sample has to be amplified by PCR many times with different molecular markers [26]–[28]. A more powerful approach [29]–[31] is the heteroduplex assay of the mitochondrial cytochrome B (Cyt b) gene (reviewed in [19]). This technique can detect differences in the DNA sequences of this gene among species by differences on electrophoretic profiles [29]. Although it is increasingly used in triatomines, standardization is often difficult, and when insects have fed on more than one species the application and the analysis could be complex.

Recently, high-resolution melting (HRM) has become a sensitive genotyping method, based on the characteristics of thermal denaturation of the amplicons. This method has a much higher performance information never before achieved by classical DNA melting curve analysis [32]. HRM is performed using a fluorescent double-stranded DNA dye that can be used in fully saturating conditions [33], [34]. The amplicon is analyzed by gradual denaturation through increasing temperature and decreased fluorescence caused by the release of intercalating dye from DNA. The melting temperature (Tm) and specific shape of the melting curve result from the DNA sequence, GC content, and amplicon length [35], [36], so it is a useful technique to obtain species-specific genotypes. Due to the increased demand for rapid, economic, easy, and high-throughput genotyping analyses, there has been a considerable focus on HRM, which can detect sequence variants without sequencing or hybridization procedures [37]–[40]. Here we present a fast, inexpensive, accurate, and sensitive technique to identify blood-meal sources of triatomines based on Cyt b gene HRM analysis of host-specific genotypes and its application in field studies.

## Materials and Methods

### Ethics statement

All Animals were handled in strict accordance with good animal practice as defined by the Colombian code of practice for the care and use of animals for scientific purposes. Ethical approval (Act N° 53, 30/06/2009) for analyzing animal specimens was obtained from the animal ethics Committee of the University of Antioquia, Medellin, Colombia.

### Identification of HRM-Cyt b profiles in standard samples

To differentiate among species-specific genotypes of natural blood-meal sources of triatomine bugs, a 383-bp fragment from the Cyt b gene was amplified from DNA samples extracted from tissue or blood of 36 individuals corresponding to 14 species. Species were chosen according to their epidemiological relevance as reservoirs of *T. cruzi* or by their proximity to anthropic environments. The species, number of individuals per species, and type of sample used to obtain DNA are shown in Table 1.

**Table 1:** Source of the sample of the standard species used in the HRM analysis.

Standard species	Common name	Tissue	blood	Total
*Didelphis marsupialis*	Opossum	-	3	3
*Mus musculus L.*	Mouse	1	2	3
*Bos taurus*	Cow	2	1	3
*Capra aegagrus*	Goat	-	3	3
*Canis lupus familiaris*	Dog	-	3	3
*Ovis aries*	Sheep	-	1	1
*Equus caballus*	Horse	-	3	3
*Sus scrofa*	Pig	3	-	3
*Oryctolagus cuniculus*	Rabbit	-	2	2
*Felis catus*	Cat	-	3	3
*Rattus norvegicus*	Rat	-	1	1
*Equus asinus*	Donkey	-	2	2
*Homo sapiens L.*	Human	-	3	3
*Gallus gallus L.*	Chicken	2	1	3

-: sample not used.

### Validation of HRM-Cyt b profiles in samples from triatomines

To validate HRM-Cyt b profiles, six fifth-stage nymphs per species of *Rhodnius prolixus*, *R. colombiensis*, and *Triatoma maculata* maintained under controlled laboratory conditions of temperature (28°C) and relative humidity (70%) were fed with chicken blood until satiated. Feces from three individuals of each species and intestinal content from three other individuals were collected 5 days post-feeding for posterior DNA extraction and amplification of the Cyt b gene. Additionally, the sensitivity of HRM Cyt b over time was analyzed in two groups of five *R. prolixus* adults fed with chicken or human blood. The intestinal content and feces were collected 1, 5, 15, and 30 days post-feeding and processed as below.

To evaluate the capability of this technique to detect HRM-Cyt b profiles in mixed feeds, five fifth-instar *R. prolixus* individuals were fed with both mice and chicken blood and the intestinal content and feces were collected at 5 days post-feeding for subsequent HRM analysis.

### Application of the HRM Cyt b technique to identify blood-meal sources from field triatomines

To apply the HRM technique to triatomines from the field, a total of 20 insects were collected in four localities of Colombia's Caribbean region, which display different eco-epidemiological transmission cycles of *T. cruzi*, as follows: (1) six domiciliary *R. prolixus* and three peridomiciliary *T. dimidiata* were collected by timed manual capture in an indigenous community with high epidemiological risk from the Sierra Nevada de Santa Marta area [41]; (2) three peridomiciliary *T. maculata* and one sylvatic *Eratyrus cuspidatus* (light-trap captured) were collected in the town of Talaigua Nuevo, located in the Bolivar department where a moderate epidemiological risk exists [42]; (3) three sylvatic *T. dimidiata* were collected manually in a hole in a fallen tree in Turbo, near the border between Colombia and Panama in the Antioquia department; this region is considered a nonendemic zone and a few reports on triatomine visiting houses have been released [43]; (4) finally, four sylvatic *R. pallescens* were collected in a palm tree in Aguachica in the Cesar department, where nondomiciliary triatomines have been reported [43]; they were caught using live chicken-bait traps (Table S1).

All insects were placed in plastic bottles, marked, transported to the laboratory, and identified according to the classification proposed by Lent and Wygodzinsky [44]. Feces samples from insects were collected and analyzed by HRM of the Cyt b gene to identify the blood-meal sources of each insect.

### DNA extraction

DNA was extracted using the phenol-chloroform method [45], with some modifications. Briefly, 200 µL of serum, 25 mg of tissue, 25 µL of intestinal content, or 30 µL of feces were incubated at 37°C for 4 h with 200 µg of proteinase K, 1% sodium dodecyl sulfate, 2.5 mM disodium EDTA, and 25 mM sodium acetate. DNA was extracted with phenol and chloroform, precipitated with 0.3 M sodium acetate and absolute ethanol, washed with 70% ethanol, vacuum dried, and then dissolved in 50 µL of distilled water. Quality and DNA concentration were measured using a Nanodrop 2000 spectrophotometer (NanoDrop Technologies, Wilmington, DE, USA).

### Amplification of the Cyt b gene using real-time PCR

A 383-bp fragment from the Cyt b gene was real time PCR-amplified from genomic DNA samples using the 5′-CCCCTCAGAATGATATTTGTCCTCA-3′ and 5′- CCATCCAACATCTCAGCATGATGAAA-3′ primers [29]. Real time PCR was performed at a final volume of 10 µL, containing 0.5 µM of each primer, 0.25 mM of each dNTP, 3.5 mM MgCl_2_, 0.5 U TrueStart Hot Start polymerase, 1.5× SYBR green dye (Invitrogen), and 10 ng of genomic DNA. Each sample was amplified in triplicate. The thermal cycling conditions were as follows: pre-heating at 95°C for 5 min, 35 cycles at 95°C for 30 s, 60°C for 30 s, and 72°C for 30 s in a thermal cycler Rotor Gene Q system (Qiagen). Following the real time PCR, melting-curve analysis of amplicons was conducted in the thermocycler (Rotor-Gene Q) by increasing the temperature from 72°C to 92°C at ramping increments from 0.1°C/s, recording changes in fluorescence with changes in temperature (dF/dT), and plotting against changes in temperature. DNA extracted from triatomine legs and *T. cruzi* were used as negative control.

### High-resolution melt curve analyses

HRM analysis was carried out using the Rotor Gene Q software v2.2 with normalization regions between 76.15–78.65°C and 89.50–91.00°C. Genotypes were defined by selecting a sample from each standard species as a reference control to identify unknown samples. The software then auto-called the genotype and melting temperatures of each amplicon and provided a confidence percentage based on the square root of the correlation coefficient between samples and the reference genotypes. Any specimen generating melt curves but having a dF/dT less than 1.0 was considered not subjected to genotyping. Averages of melting temperatures and the confidence percentage of the specimen replicates were assigned to a representative genotype, and the standard deviation, confidence intervals, and variation coefficient were calculated using the Prism GraphPad v4.0 software (GraphPad software, Inc).

### Sequencing

To confirm the sequence identity of those samples tested by HRM analysis, a 358-bp fragment of Cyt b was sequenced for the standard samples and ten test samples chosen randomly (2, 3, 4, 5, 10, 12, 15, 16, 19, and 20 (Table S1)). For each specimen, both forward and reverse sequences were used to generate a consensus sequence using Bioedit v. 7.0.5 [46], and then positional nucleotide sites were compared after multiple alignment done using the ClustalW algorithm [47] implemented in Bioedit v. 7.0.5 [48]. Additionally, a nucleotide Basic Local Alignment Search (Blastn) was performed to estimate the matched hit scores, identity percentages and e-values of the Cyt b tested. Finally, the distance tree based on net genetic distances (p-distances) was performed using the neighbor-joining algorithm with 1000 bootstrap replicates using MEGA 5.05 [49].

## Results

### Genotyping of standard samples using HRM analysis of the Cyt b gene

**Figure 1:**
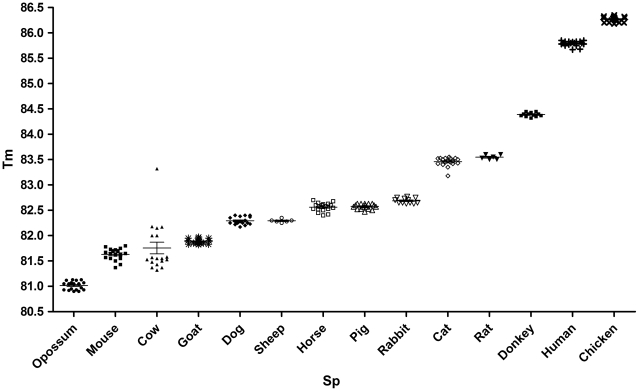
Tm distribution of 14 genotypes included in the study. Tm: melting temperature, Sp: species.

**Figure 2:**
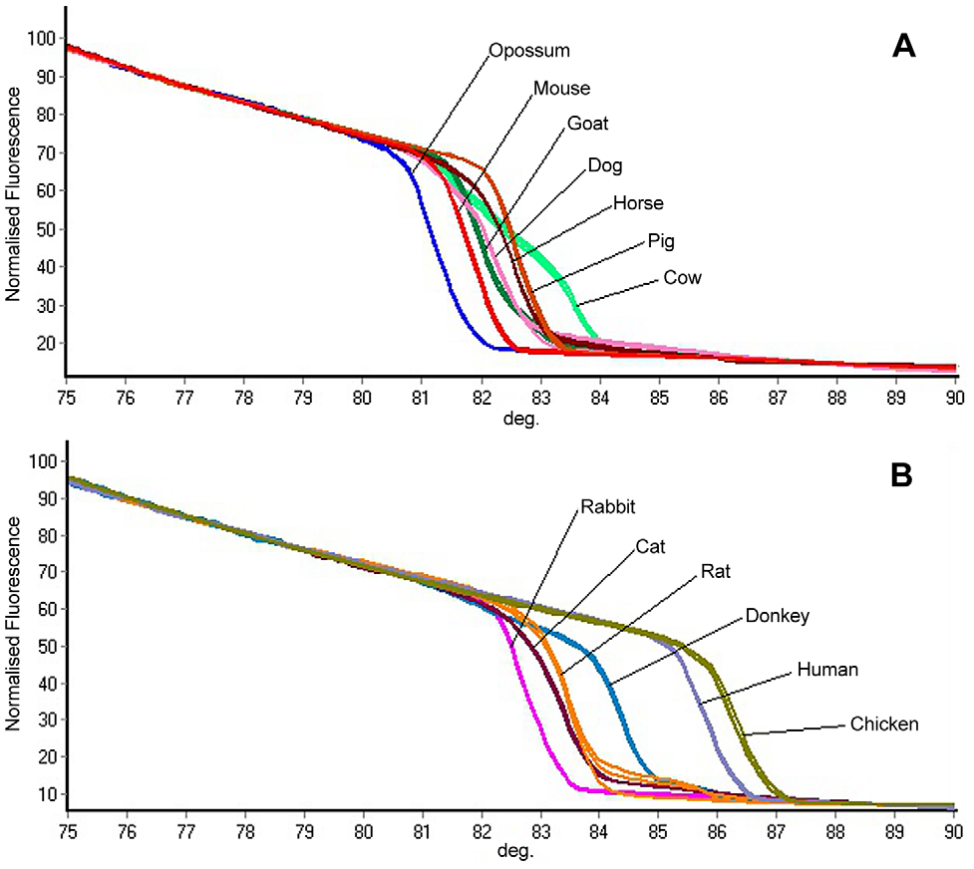
Dissociation curve of 13 of the 14 genotypes included in the study.

All samples analyzed amplified a 383-bp product, as previously reported to the Cyt b gene [29] (data not shown) and no product was obtained when the insect DNA or *T. cruzi* DNA were used. The amplicon melt curve and Tm showed low intraspecies variability (Table 2, Figure 1). A specific profile for each species analyzed was observed (Figures 2 and S1), with confidence percentages (%C) ranging from 78.81% and 98.69% (Table 2), indicating that samples were correctly identified in each of the experiments conducted in triplicate.

**Table 2:** Fourteen standard species included in the study and its respective Tm and HRM analysis.

	Tm analysis					HRM analysis		
Sp	Tm	SD	95% CI of Tm	VC	Tm genotype	HRM genotype	%C	%C SD
Opossum	81.02	0.09	80.97–81.06	0.10	Opossum	Opossum	81.24	15.02
Mouse	81.63	0.12	81.57–81.69	0.14	Mouse	Mouse	94.19	4.94
Cow	81.76	0.49	81.51–82.00	0.60	Cow	Cow	83.07	16.63
Goat	81.88	0.05	81.86–81.91	0.06	Goat	Goat	88.24	13.25
Dog	82.29	0.08	82.25–82.33	0.09	Dog	Dog	90.92	13.86
Sheep	82.29	0.03	82.26–82.33	0.04	Sheep	Sheep	98.69	1.50
Horse	82.56	0.08	82.52–82.60	0.10	Horse	Horse	93.46	9.04
Pig	82.58	0.05	82.55–82.60	0.06	Pig	Pig	95.18	9.50
Rabbit	82.69	0.05	82.66–82.72	0.06	Rabbit	Rabbit	98.55	1.33
Cat	83.46	0.09	83.41–83.50	0.11	Cat	Cat	78.81	22.40
Rat	83.55	0.05	83.50–83.59	0.05	Rat	Rat	89.90	6.51
Donkey	84.39	0.04	84.36–84.41	0.05	Donkey	Donkey	87.85	22.14
Human	85.79	0.05	85.76–85.82	0.06	Human	Human	96.58	2.22
Chicken	86.27	0.05	86.24–86.29	0.06	Chicken	Chicken	93.05	18.01

SD: standard deviation, CI: confidence interval, VC: variability correlation, %C: confidence percentage, %C SD: standard deviation of confidence percentage, Tm Genotype: Genotype according to Tm from all the replicas.

Although some species exhibited the same Tm value, HRM profiles in these species were clearly discriminated and recognized as different genotypes, such as dog and sheep or pig and horse (Table 2, Figure S1). On the other hand, although the cow melt curve showed two peaks, the genotype was recognized with a high confidence percentage (Table 2). These two peaks were found in different types of samples (muscle and blood).

### Validation of the HRM technique to identify blood-meal sources in triatomines

#### Analysis of intestinal contents and feces from different species of insects

The HRM profile of the Cyt b gene obtained from feces and intestinal content from *R. colombiensis*, *R. prolixus*, and *T. maculata* fed with chicken blood were analyzed and classified based on the genotypes defined with the standard samples. All samples were correctly classified as blood-fed chicken (C) and just one of the samples obtained from feces (*R. prolixus*) showed low confidence percentage. Table S2 summarizes the results of the amplifications obtained for each of these species.

#### Analysis of intestinal content and feces at different times post-feeding and after mixed feeding

All samples were successfully amplified by qPCR 1, 5, 15, and 30 days post-feeding (Table 3). Intestinal content and feces samples showed successful amplification and detection of the blood-meal source after 30 days post-feeding, indicating the high sensitivity of Cyt b-HRM at this time. Interestingly, the samples from insects fed on humans showed one peak classified as a chicken genotype at 1 and 5 days post-feeding. This result is not surprising considering that insects were maintained in laboratory conditions with chicken blood meal before feeding with human blood. However, after 15 days post-feeding, a second peak appeared which corresponded to the human genotype (Table 3).

**Table 3:** Samples from intestinal content and feces at different times after feeding.

			Tm Analysis						HRM Analysis		
Feed	Sample Source	Time course(days)	Tm1	SD	Tm1 Genotype	Tm2	SD	Tm2 Genotype	HRM Genotype	HRM %C	%C SD
H	IC	1	86.24	0.02	CG	-	-	-	CG	55.23	10.84
H	IC	5	86.28	0.03	CG	-	-	-	CG	89.49	8.73
H	IC	5	86.3	0.03	CG	-	-	-	CG	82.15	8.77
H	IC	15	81.17	0.06	M	85.89	0.06	H	ND	ND	ND
H	IC	15	81.43	0.04	M	86.47	0.02	C	ND	ND	ND
H	IC	30	86.26	0.01	CG	-	-	-	CG	61.32	3.10
H	IC	30	85.79	0.01	HG	-	-	-	HG	63.65	5.66
H	F	30	81.54	0.01	M	85.97	0.03	H	ND	ND	ND
H	F	30	85.9	0.05	HG	-	-	-	HG	21.75	1.93
C	IC	1	86.32	0.03	CG	-	-	-	CG	94.49	3.60
C	IC	1	86.32	0.03	CG	-	-	-	CG	96.83	1.80
C	IC	1	86.28	0.02	CG	-	-	-	CG	89.45	5.88
C	IC	5	86.34	0.01	CG	-	-	-	CG	93.85	1.11
C	IC	5	86.34	0.04	CG	-	-	-	CG	95.56	4.01
C	IC	15	86.35	0.00	CG	-	-	-	CG	97.11	1.80
C	IC	15	86.31	0.01	CG	-	-	-	CG	96	2.75
C	IC	30	86.34	0.04	CG	-	-	-	CG	91.93	8.59
C	IC	30	86.32	0.02	CG	-	-	-	CG	98.37	0.05
C	F	30	86.41	0.05	CG	-	-	-	CG	33.37	18.35

H: human, C: chicken, IC: intestinal content, F: feces, CG: chicken genotype, HG: human genotype, M: mixed feeding determined by two peaks in the melting curve, Tm1: melting temperature of peak 1, Tm2: melting temperature of peak 2, SD: standard deviation, HRM %C: confidence percentage HRM, %C SD standard deviation of confidence percentage, ND: not determined, -: No secondary peak.

Therefore, we evaluated the possibility of detecting at least two different blood meal sources. In this case, the melt curve showed two peaks belonging to mouse and chicken amplicons, with their respective Tm. In addition, the dissociation curve had a shape with two falls (Figure S2). These types of curves were analyzed by modifying the normalization area to every fall of the curve to recognize genotypes separately with their respective standard genotype.

### Identification of blood sources in triatomines from the field

**Figure 3:**
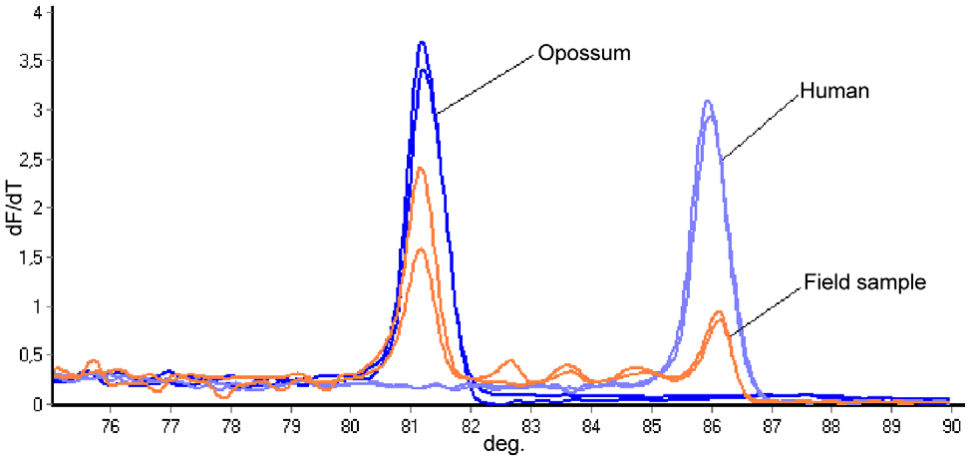
Field sample showing mixed feeding including opossum and human with their respective controls.

The HRM analysis of Cyt b in *R. prolixus* collected inside dwellings indicated that five of the six individuals evaluated had at least a human blood-meal source, and the remaining one a dog blood-meal source (Table 4). Two peridomiciliary *T. dimidiata* showed mixed blood-meal sources (human and dog; human and a nonidentified source) (Table 4). Sylvatic *T. dimidiata* showed a mixed blood-meal source of opossum, human, and a nonidentified source. Of two *T. maculata* caught in a house, one was found to have fed on a human blood-meal source, while the other had a mixed human and nonidentified source. Finally, the sylvatic *E. cuspidatus* and *R. pallescens* were identified as being blood-fed from human, chicken, and nonidentified sources (Table 4, Figure 3).

**Table 4:** Amplification results from field samples (Table S1) showing one or two peaks in the melting curve.

	Lowest temperature peak analysis						Highest temperature peak analysis					
Sample	Tm1	SD	Tm1 genotype	HRM genotype	HRM %C	SD	Tm2	SD	Tm2 genotype	HRM genotype	HRM %C	SD
1	86.07	0.07	Human	Human	83.29	14.60	-	-	-	-	-	-
2	85.96	0.06	Human	Human	90.98	5.02	-	-	-	-	-	-
3	85.96	0.05	Human	Human	81.84	6.48	-	-	-	-	-	-
4	86.15	0.06	Human	Human	75.41	2.02	-	-	-	-	-	-
5	86.03	0.04	Human	Human	90.91	3.56	-	-	-	-	-	-
6	82.04	0.08	Dog	Dog	56.48	3.30	-	-	-	-	-	-
7	82.02	0.15	Dog	Dog	53.68	25.81	86.14	0.04	Human	Human	64.56	14.75
9	84.74	0.02	ND	ND	ND	ND	86.13	0.03	Human	Human	63.11	9.50
10	84.87	0.21	ND	ND	ND	ND	86.10	0.04	Human	Human	78.90	15.17
12	81.16	0.01	Opossum	Opossum	86.02	11.19	86.10	0.03	Human	Human	82.24	3.30
14	86.15	0.02	Human	Human	56.35	6.93	-	-	-	-	-	-
15	84.85	0.09	ND	ND	ND	ND	86.20	0.06	Human	Human	40.76	11.44
16	86.19	0.06	Human	Human	57.23	11.38	-	-	-	-	-	-
19	84.85	0.08	ND	ND	ND	ND	86.16	0.01	Human	ND	ND	ND
20	86.58	0.02	Chicken	Chicken	88.71	7.08	-	-	-	-	-	-

Tm1: melting temperature of peak 1, Tm2: melting temperature of peak 2, SD: standard deviation, HRM %C: confidence percentage of HRM analysis, ND: not determined or not recognized, -: No secondary peak.

As mentioned above, four mixed samples, including one intradomiciliary insect, showed a peak with a Tm = 84.83±0.05, which was inconsistent with any of our standards (Table 4), indicating another additional blood-meal source that was not considered in this study. Five samples did not amplify.

### Sequence analysis of blood sources in triatomines confirmed the HRM result

Blastn results showed significant identity values greater than 95% for all samples except for sample number 20 (Table 5). Multiple alignment of the test sample and standards showed great nucleotide similarity and coherence with HRM results previously obtained. The percentage of identity among the field-collected samples and their respective standards ranged between 95% and 99% in samples from human and opossum, and 57% in the sample from chicken (Table 5). Finally, the distance tree showed well-supported groups according to HRM results (Figure S3). It is worth mentioning that the field-collected samples sequenced included a sample (number 12) that was positive for mixed feeding for HRM analysis. In this case, the sequence analysis also identified the two species (human (12_1) and opossum (12_2)) (Table 5).

**Table 5:** Blastn results of ten insects collected from the field.

Sample	Blastn description	e-Value	Accessionnumber	Identity with the standard used to HRM identification
2	*H. sapiens*	0	AY509658.1	98.30%
3	*H. sapiens*	0	AY509658.1	98.32%
4	*H. sapiens*	0	AY509658.1	98.89%
5	*H. sapiens*	0	AY509658.1	98.32%
10	*H. sapiens*	0	AY509658.1	98.89%
12_1	*H. sapiens*	2.00E-161	AY509658.1	94.69%
12_2	*D. marsupialis*	2.00E-167	DQ236278.1	98.60%
15	*H. sapiens*	0	AY509658.1	98.89%
16	*H. sapiens*	9.00E-175	HQ384199.1	97.49%
19	*H. sapiens*	9.00E-180	AY509658.1	98.04%
20	*G. gallus*	4.00E-33	FM205717.1	59.78%

For each sample the identity with the standard species used for HRM identification is shown.

## Discussion

The epidemiological scenario of Chagas disease has become increasingly complex over the years. The natural habitats of some human populations within the forest and deforestation caused by humans are but two of the reasons that may complicate this scenario. Thus, the classical separation of transmission cycles defined for this disease could be different for many places, making it difficult to determine the epidemiological characteristics of particular regions. With this unclear scenario, the determination of blood-meal sources in hematophagous vectors has become essential to surveillance and prevention of potential infection foci.

In this study, HRM analysis of the Cyt b gene made it possible to identify 14 species successfully even when some of them had the same Tm values. Each species was well recognized under a variety of species reaching high confidence percentage values, thus showing the power of this classification using gene amplification and HRM analysis. It is worth noting that this recognition is possible with a single PCR, making it a quick and inexpensive technique.

It is important to highlight that no studies with this number of species standards have been conducted with Chagas disease vectors. Pizarro and Stevens [28] designed primers against different targets from 11 species and Mota et al. [26] also identified 11 species in their study. ELISA-based techniques could identify the same number of species, but it is very expensive and, in most cases, anti-serum for many species is not commercially available. On the other hand, studies based on PCR methods may also increase the number of species, but it is necessary to standardize PCR conditions for each species. Additionally, every DNA sample must be submitted to a number of PCRs to identify the blood sources, making the technique very expensive. Instead, the technique used herein only needs a single pair of primers, making it easy to include other species according to every epidemiological scenario with no other standardizations of PCR conditions.

A similar power is reached when DNA extracted from intestinal content and feces is analyzed with this technique, demonstrating that both can be used for the identification of the blood source from insects. Although no misidentification was recorded with DNA from insect feces, low confidence percentages were reached and other samples did not amplify, showing that the quality of the sample is a limitation of the technique, as reported for real-time PCR [50]. Nonetheless, work with feces has substantial advantages because it is sometimes hard to extract intestinal content from insects, especially when they have been starved for a long time. Another benefit of feces is the possibility of making a diagnosis of *T. cruzi* infection and the determination of the blood-meal source; both tests that can be done with a single sample of DNA without sacrificing the insect.

The HRM technique proved to work in both types of samples (intestinal and fecal) at least until 30 days after insects were fed. This showed the applicability of the technique even when the blood-meal sources were limited to the triatomines. Pizarro and Stevens [28] reported on the feasibility of their technique, based on the primer design for each species, for 2 months. It is probable that our technique has identical results, but this was not verified. However, many factors have an influence on the presence of blood in the intestinal tract over time, as described by Lee et al. [30]. Chicken blood has nucleated erythrocytes, so a large amount of DNA should remain in the intestine for a long time. This could explain the presence of unspecific peaks in the melt curve when insects were fed with human blood because the insects are usually fed with chicken blood in laboratory conditions. Thus, the present results suggest that chicken blood remains in the intestinal tract longer than mouse and human blood (data not shown).

On the other hand, the cow profile showed two melting peaks, suggesting the amplification of nonspecific fragments. This could stem from nuclear copies of mtDNA (Numts). It is well known that Numts can be amplified in genetic studies based on mtDNA [51], [52]. Some of these Numts have been described in cattle [53]. However, this phenomenon does not produce mistakes in classification because the shape remains the same across different specimens included.

Unspecific peaks led to testing mixed feedings, showing that the technique detects both Tm sources. This allows identification of many species from which the insects have been fed, even when there are no standards for those species. This is impossible with methods such as ELISA or techniques based on PCR-specific primers. Heteroduplex analysis identifies mixed feeding as well, but the electrophoretic profile becomes very complex, making it difficult to analyze, and sometimes it does not recognize even a single species within the mixture [30]. Sequencing of DNA fragments can resolve mixed feedings well [54], but is quite difficult to analyze and time-consuming. However, we suggest using sequencing of amplicons only when unidentified samples by HRM analysis are present.

We processed 20 samples from the field but only 15 amplified. The other five samples were probably of insufficient quality, which is not surprising because it was feces DNA.

In general, the expected genotypes were recognized by the analysis. Those insects captured inside houses showed human feeding and one dog feeding, suggesting a domestic transmission cycle involving humans and to a lesser extent dogs, which has been reported to have epidemiological relevance [55]–[58]. Samples from the peridomestic or sylvatic cycle also showed human feeding, in agreement with other studies showing that *R. prolixus* and *T. dimidiata* fed mostly on human blood [23]. Sometimes, mixed feeding with human blood showed a low peak in the melt curve (Figure 3). It is possible that the other techniques were not sensitive enough to detect human blood because of the small amount of DNA in the sample [31].

We included *R. pallescens* caught with live-bait trap, which showed chicken-blood feeding as expected by the type of bait used. This control from the field confirms the capacity and accuracy of the technique even when field samples are analyzed, which is the aim of technique.

*E. cuspidatus* showed human feeding, which is surprising because is a strictly sylvatic species. However, Dib et al. [59] incriminated *E. cuspidatus* in transmission of parasite when comparing RAPD profiles from *T. cruzi* strain isolates from human and vector. These two results showed that although sylvatic this species probably visits dwellings looking for feeding sources. Additionally, Cortés and Suárez [42] reported *E. cuspidatus* inside houses in the study area, suggesting that the insect occasionally colonized intra- and peridomestic environments, which is confirmed by the present result. More studies must be conducted to determine the role of this species in transmission of *T. cruzi* in this area.

There was only one genotype that could not be identified with a Tm = 84.83°C±0.05, indicating that the number of species standards included in the study was adequate. However, this could be improved knowing the fauna and diversity of species of a particular study area. When the sample is not recognized, the next step is sequencing, as reported by other authors [29]–[31]. Based on these results, we recommend the use of Tm values as a first approach to identify a sample from insects collected in natural populations. Then the species selected based on the Tm could be used as a standard sample for HRM analysis, and finally the species not identified could be sequenced.

In conclusion, we believe that epidemiological studies involving vectorial incrimination and transmission dynamics must identify the blood-meal source to cover the entire panorama of transmission. HRM analysis of the Cyt b gene is the most powerful technique in this type of study because it can accurately identify the species even when the vector has mixed feeding, it has a high resolution power, and it is fast, easy, and inexpensive. However, it is important to obtain high-quality DNA and be mindful of the fauna of the study area to have an adequate number of standard species.

## Supporting Information

Figure S1 **Discrimination of genotypes with similar Tm.** A: sheep and dog genotypes with Tm of 82.29 for both. B: horse and pig genotypes with Tm of 82.56 and 82.58, respectively.
(TIF)

Figure S2 **Dissociation curve of DNA sample from insect with mixed feeding with mouse (Tm = 81.63) and chicken (Tm = 86.27).** Arrows indicate falls of the curve indicating each genotype.
(TIF)

Figure S3 **Distance tree based on net genetic distances (p-distances) with neighbor-joining algorithm with 1000 bootstrap replicates.** *Gavialis gangeticus* was used as outgroup.
(PDF)

Table S1 **Field samples included in this study and geographic distribution.** Location corresponds to locality followed by the department. Ecotope corresponds to the place where the bugs were collected. SNSM: Sierra Nevada de Santa Marta.
(DOCX)

Table S2 **Tm and HRM analysis from DNA samples from feces and intestinal content of three triatominae species.** IC: Intestinal content, F: Feces; Tm: Melting Temperature, %C: confidence percentage, %C SD: Standard deviation of confidence percentage, C: Chicken.
(DOCX)
