# Supplementary figures and images for: High-Resolution Melting (HRM) of the Cytochrome B Gene: A Powerful Approach to Identify Blood-Meal Sources in Chagas Disease Vectors
Source: PLoS Negl Trop Dis. 2012 Feb 28;6(2):e1530. doi: 10.1371/journal.pntd.0001530 (PMC3289613; doi:10.1371/journal.pntd.0001530)

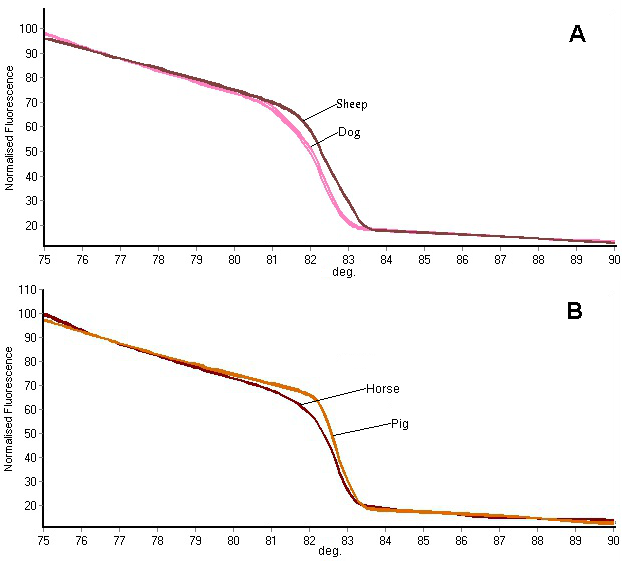

Supplement: Figure S1 — Discrimination of genotypes with similar Tm. A: sheep and dog genotypes with Tm of 82.29 for both. B: horse and pig genotypes with Tm of 82.56 and 82.58, respectively. (TIF) [file pntd.0001530.s001.tif]

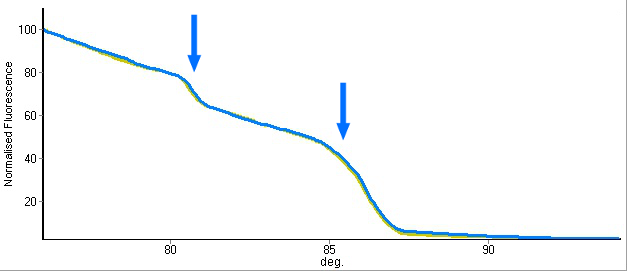

Supplement: Figure S2 — Dissociation curve of DNA sample from insect with mixed feeding with mouse (Tm = 81.63) and chicken (Tm = 86.27). Arrows indicate falls of the curve indicating each genotype. (TIF) [file pntd.0001530.s002.tif]

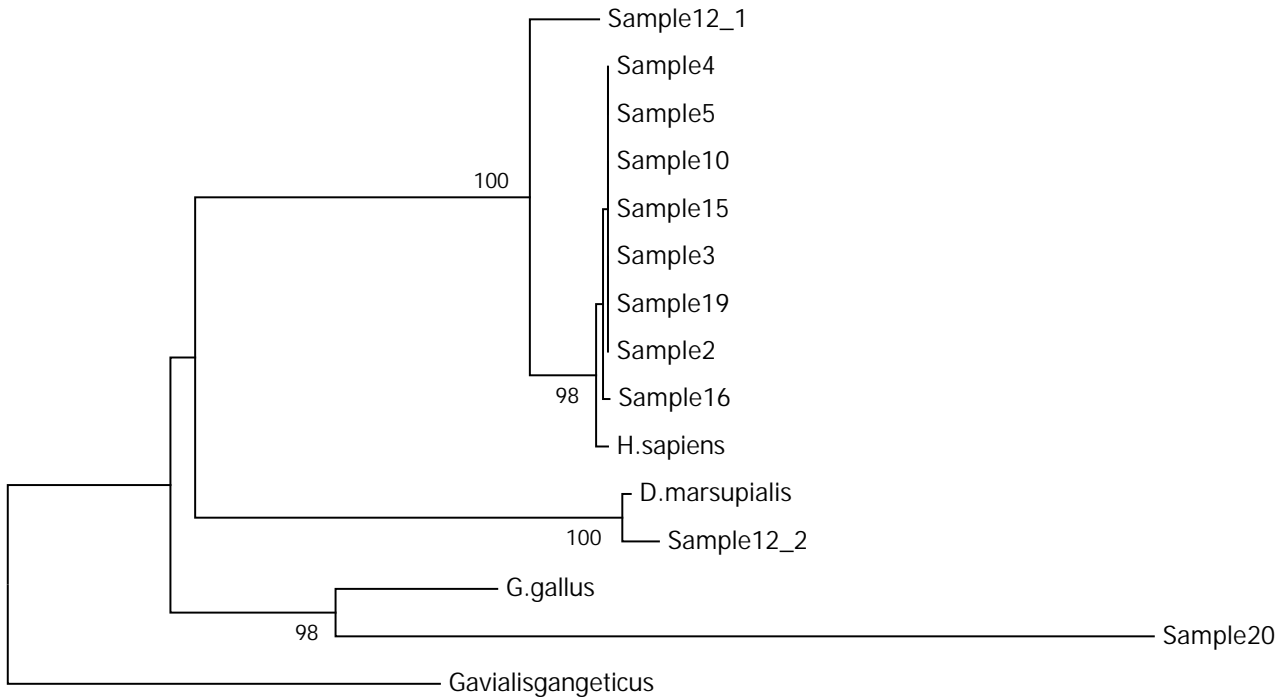

0.05

Supplement: Figure S3 — Distance tree based on net genetic distances (p-distances) with neighbor-joining algorithm with 1000 bootstrap replicates. Gavialis gangeticus was used as outgroup. (PDF) [file pntd.0001530.s003.pdf]
