# Supplementary material for: High-Resolution Melting (HRM) of the Cytochrome B Gene: A Powerful Approach to Identify Blood-Meal Sources in Chagas Disease Vectors
Source: PLoS Negl Trop Dis. 2012 Feb 28;6(2):e1530. doi: 10.1371/journal.pntd.0001530 (PMC3289613; doi:10.1371/journal.pntd.0001530)
Supplement: Table S1 — Field samples included in this study and geographic distribution. Location corresponds to locality followed by the department. Ecotope corresponds to the place where the bugs were collected. SNSM: Sierra Nevada de Santa Marta. (DOCX) [file pntd.0001530.s004.docx]

**Table S1.** **Field samples included in this study and geographic distribution.**

| **Sample** | **Specie** | **Location** | **Ecotope** |
| --- | --- | --- | --- |
| **1** | *R. prolixus* | SNSM, La Guajira | Domestic |
| **2** | *R. prolixus* | SNSM, La Guajira | Domestic |
| **3** | *R. prolixus* | SNSM, La Guajira | Domestic |
| **4** | *R. prolixus* | SNSM, La Guajira | Domestic |
| **5** | *R. prolixus* | SNSM, La Guajira | Domestic |
| **6** | *R. prolixus* | SNSM, La Guajira | Domestic |
| **7** | *T. dimidiata* | SNSM, La Guajira | Peridomiciliary |
| **8** | *T. dimidiata* | SNSM, La Guajira | Peridomiciliary |
| **9** | *T. dimidiata* | SNSM, La Guajira | Peridomiciliary |
| **10** | *T. dimidiata* | Turbo, Antioquia | Sylvatic |
| **11** | *T. dimidiata* | Turbo, Antioquia | Sylvatic |
| **12** | *T. dimidiata* | Turbo, Antioquia | Sylvatic |
| **13** | *T. maculata* | Talaigua Nuevo, Bolívar | Peridomiciliary |
| **14** | *T. maculata* | Talaigua Nuevo, Bolívar | Peridomiciliary |
| **15** | *T. maculata* | Talaigua Nuevo, Bolívar | Peridomiciliary |
| **16** | *E. cuspidatus* | Talaigua Nuevo, Bolívar | Sylvatic |
| **17** | *R. pallescens* | Aguachica, Cesar | Sylvatic |
| **18** | *R. pallescens* | Aguachica, Cesar | Sylvatic |
| **19** | *R. pallescens* | Aguachica, Cesar | Sylvatic |
| **20** | *R. pallescens* | Aguachica, Cesar | Sylvatic |

Location corresponds to locality followed by the department. Ecotope corresponds to the place where the bugs were collected. SNSM: Sierra Nevada de Santa Marta.
