# Supplementary material for: High-Resolution Melting (HRM) of the Cytochrome B Gene: A Powerful Approach to Identify Blood-Meal Sources in Chagas Disease Vectors
Source: PLoS Negl Trop Dis. 2012 Feb 28;6(2):e1530. doi: 10.1371/journal.pntd.0001530 (PMC3289613; doi:10.1371/journal.pntd.0001530)
Supplement: Table S2 — Tm and HRM analysis from DNA samples from feces and intestinal content of three triatominae species. IC: Intestinal content, F: Feces; Tm: Melting Temperature, %C: confidence percentage, %C SD: Standard deviation of confidence percentage, C: Chicken. (DOCX) [file pntd.0001530.s005.docx]

**Table S2**. **Tm and HRM analysis from DNA samples from feces and intestinal content of three triatominae species**.

| **Specie** | **Sample source** | **Number of samples** | **Tm average** | **HRM Genotype** | **%C Average** | **%C SD** |
| --- | --- | --- | --- | --- | --- | --- |
| *R. colombiensis* | IC | 3 | 86.08 | C | 91.83 | 3.29 |
| *R. colombiensis* | F | 3 | 86.07 | C | 84.81 | 7.37 |
| *R. prolixus* | IC | 3 | 85.99 | C | 85.46 | 12.98 |
| *R. prolixus* | F | 3 | 86.39 | C | 86.57 | 6.57 |
| *T. maculata* | IC | 3 | 86.04 | C | 90.32 | 6.15 |
| *T. maculata* | F | 3 | 86.27 | C | 51.03 | 7.41 |

IC: Intestinal content, F: Feces; Tm: Melting Temperature, %C: confidence percentage, %C SD: Standard deviation of confidence percentage, C: Chicken
